# Human T-cell lymphotropic virus type 1 (HTLV-1) grip on T-cells: investigating the viral tapestry of activation

**DOI:** 10.1186/s13027-024-00584-5

**Published:** 2024-05-11

**Authors:** Arash Letafati, Atefeh Bahavar, Alijan Tabarraei, Mehdi Norouzi, Abdollah Amiri, Sayed-Hamidreza Mozhgani

**Affiliations:** 1 Department of Virology, School of Public Health, Tehran University of Medical Sciences, Tehran, Iran; 2 Research Center for Clinical Virology, Tehran University of Medical Science, Tehran, Iran; 3 Department of Microbiology, School of Medicine, Golestan University of Medical Sciences, Gorgan, Iran; 4 Department of Microbiology and Virology, School of Medicine, Alborz University of Medical Sciences, Karaj, Iran

**Keywords:** HTLV-1, T-cell activation, mRNA expression, HAM/TSP, ATLL

## Abstract

**Introduction:**

Human T-cell Lymphotropic virus type 1 (HTLV-1) belongs to *retroviridae* which is connected to two major diseases, including HTLV-1-associated myelopathy/tropical spastic paraparesis (HAM/TSP) and Adult T-cell leukemia/lymphoma (ATLL). This study aims to investigate the mRNA expressions of key proteins correlated to T-cell activation in asymptomatic carriers (ACs) HTLV-1 infected patients, shedding light on early molecular events and T-cell activation following HTLV-1 infection.

**Material and Methods:**

The study involved 40 participants, including 20 ACs and 20 healthy subjects. Blood samples were collected, ELISA assessment for screening and confirmation with PCR for Trans-activating transcriptional regulatory protein (Tax) and HTLV-1 basic leucine zipper factor (HBZ) of the HTLV-1 were done. mRNA expressions of C-terminal Src kinase (CSK), Glycogen Synthase Kinase-3 Beta (GSK3β), Mitogen-Activated Protein Kinase 14 (MAP3K14 or NIK), Phospholipase C Gamma-1 (PLCG1), Protein Tyrosine Phosphatase non-Receptor Type 6 (PTPN6) and Mitogen-Activated Protein Kinase Kinase Kinase-7 (SLP-76) and Mitogen-Activated Protein Kinase14 (MAP3K7 or TAK1) were assayed using RT-qPCR. Statistical analyses were performed using PRISM and SPSS software.

**Results:**

While there were no significant upregulation in CSK and PTPN6 in ACs compared to healthy individuals, expression levels of GSK3β, MAP3K14, PLCG1, SLP-76, and TAK1 were significantly higher in ACs compared to healthy subjects which directly contributes to T-cell activation in the HTLV-1 ACs.

**Conclusion:**

HTLV-1 infection induces differential mRNA expressions in key proteins associated with T-cell activation. mRNAs related to T-cell activation showed significant upregulation compared to PTPN6 and CSK which contributed to T-cell regulation. Understanding these early molecular events in ACs may provide potential markers for disease progression and identify therapeutic targets for controlling viral replication and mitigating associated diseases. The study contributes novel insights to the limited literature on T-cell activation and HTLV-1 pathogenesis.

## Introduction

Human T-cell Lymphotropic virus type 1 (HTLV-1) is a retrovirus that belongs to *retroviridae* family and delta retrovirus genus. This virus is connected to two significant diseases; HTLV-1-associated myelopathy/tropical spastic paraparesis (HAM/TSP) which is progressive paralysis and Adult T-cell leukemia/ lymphoma (ATLL) which is a type of blood cancer [1]. It's also linked to other disorders like Sjogren's syndrome, infectious dermatitis, uveitis, and myocarditis [1, 2]. Although often asymptomatic, HTLV-1 can progress to HAM/TSP in 0.25–3% and ATLL in 4–7% of individuals [3, 4]. The virus is endemic in countries such as Japan, Iran, Brazil, and the Caribbean, with a prevalence of 0.2% to over 2% in vulnerable groups such as hemodialysis patients [2, 5]. HTLV-1 establishes lifelong infection by integrating its genome into the DNA of infected T cells, employing various strategies to induce pathogenesis and activate T lymphocytes, contributing to disease progression. The activation of T cells is a highly regulated process involving a network of signaling molecules and pathways [1]. Several key proteins play pivotal roles in modulating T-cell activation, ensuring a precise and controlled immune response. Among these proteins are C-terminal Src kinase (CSK), which negatively regulates T-cell receptor (TCR) signaling by inhibiting Src family kinases [6, 7]. Glycogen Synthase Kinase 3 Beta (GSK3β) is associated with T-cell activation by regulating the activity of many transcription factors like NFAT. Mitogen-Activated Protein Kinase Kinase Kinase 14 (MAP3K14 or NIK) is another protein which is involved in the non-canonical NF-κB pathway, crucial for T-cell survival and differentiation [8, 9]. Phospholipase C Gamma 1 (PLCG1) is a key player in TCR signaling, initiating calcium release and protein kinase C (PKC) activation [10, 11]. Protein Tyrosine Phosphatase, Non-Receptor Type 6 (PTPN6 or SHP-1) acts as a negative regulator, dephosphorylating tyrosine residues to modulate T-cell activation. SH2 Domain-Containing Leukocyte Protein of 76 kDa (SLP-76) serves as an adapter protein, connecting the TCR to downstream signaling molecules [12–14] Mitogen-Activated Protein Kinase Kinase Kinase 7 (MAP3K7 or TAK1) contributes to T-cell activation by activating MAP kinase pathways and NF-κB signaling in response to TCR engagement [15–17].

Investigating T-cell activation in the context of HTLV-1 infection is imperative for comprehending the multifaceted dynamics between the virus and the host immune system. HTLV-1 predominantly infects CD4 + T cells and relies on T-cell activation for efficient viral replication and the establishment of viral reservoirs throughout the body [18]. T-cell activation not only facilitates viral replication but also contributes to migration of activated T cells to various tissues, thereby expanding the potential reservoirs for HTLV-1. This migration plays an important role in the dissemination of the virus, contributing to its ability to persist in diverse anatomical locations. Moreover, insights into T-cell activation pathways affected by HTLV-1 infection provide opportunities to identify potential therapeutic targets. Targeting specific molecules involved in T-cell activation may offer strategies not only to control viral replication but also to modulate immune responses, potentially mitigating the severity of HTLV-1-associated diseases. Investigating the mRNA expressions related to T-cell activation in ACs of HTLV-1 is crucial for unraveling early molecular events in HTLV-1 infection. As ACs represent individuals harboring the virus without apparent symptoms, studying these gene expressions provides insights into the potential markers that could predict disease progression to conditions such as ATLL or HAM/TSP.

**Fig. 1:**
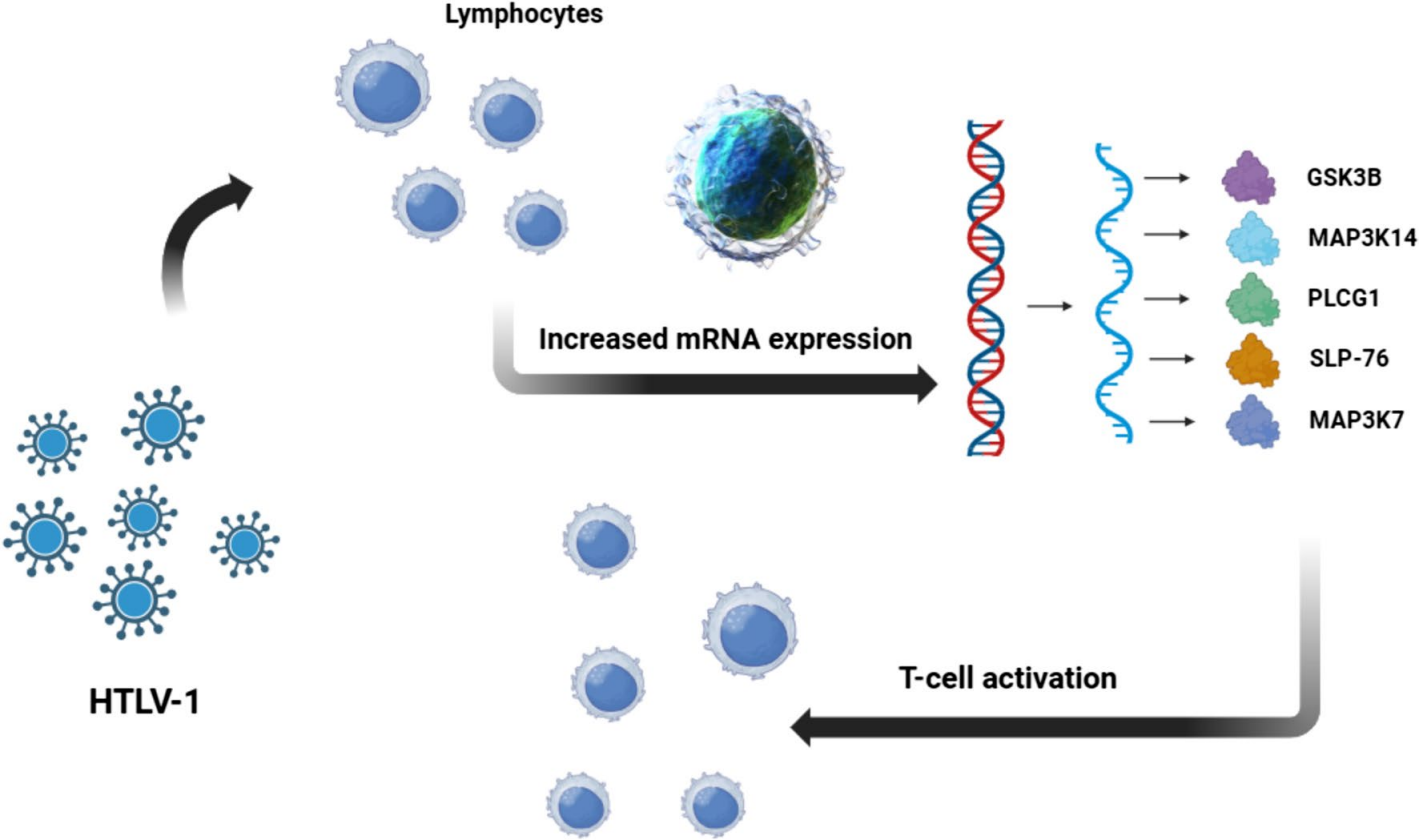
Differential mRNA Expressions of T-Cell Activation-Associated Proteins in HTLV-1 Asymptomatic Carriers (ACs) and Healthy Subjects. HTLV-1 has significant effect in the context of upregulation of GSK3β, MAP3K14, PLCG1, SLP-76, and TAK1 mRNA expressions in ACs compared to healthy individuals, shedding light on early molecular events in HTLV-1 infection and T-cell activation

In our research, we have assayed mRNA expression of GSK3β, MAP3K14, PLCG1, PTPN6, SLP-76, and TAK1 to get provision into the initial stages of HTLV-1 and its implications for disease development (Fig. 1). This project is continuous of our ongoing study on HTLV-1 which were migration and angiogenesis [2].

## Material and methods

### Study participants

This research took place at the Alborz University of Medical Sciences from 2021 to 2022, involving a total of 40 participants who referred to Alborz blood transfusion center. The participants were divided equally into two groups: 20 asymptomatic carriers (ACs) patients and 20 healthy subjects. Among those in the ACs patient group 16 males and 4 females were participated. Similarly, the healthy group had an equivalent distribution of 16 males and 4 females. On average, ACs participants average year were 48.45 years old, while healthy individuals had average of 50.30 years. All selected individuals underwent screening to confirm negative test for any other bloodborne viruses and underlying disorders such as diabetes and blood pressure.

### Sample collection

Sterile tubes containing EDTA anticoagulant were employed to collect 6 mL of blood samples, ensuring their preservation in a cold chain during transportation to the virology laboratory. The participant selection criteria for the study included individuals with asymptomatic PCR-confirmed infection for HTLV-1 and healthy subjects who had not taken any medications, had no history of autoimmune diseases, and were not currently dealing with infectious agents like Hepatitis C Virus (HCV), Human Immunodeficiency Virus (HIV) and Hepatitis B Virus (HBV). Approval for the research was granted by Tehran University of Medical Sciences in Tehran, Iran (IR.TUMS.SPH.REC.1400.319), and each participant provided signed consent.

### ELISA assessment

The ELISA test employed the DIA.PRO kit (HTLV I, II Ab version ULTRA, Italy) to identify HTLV-1 antibody in our samples. Cut-off value is also determined by measuring the light absorption of the negative control at 450 nm, and the findings were analyzed using the kit's provided instruction. Samples registering values below 0.9 were categorized as negative for HTLV-1.

### RNA extraction

Extraction of RNA from whole blood of patients and healthy individuals was performed using the RNJia Kit (ROJE, Iran) in accordance to the instructions provided by manufacturer to ensure RNA purity. In the process of cDNA synthesis, 1 µl of the random hexamer primers and also 5 µl of the extracted RNA were employed, adhering to the instructions provided by the manufacturer of the RT-ROSET Kit (ROJE, Iran). RNA concentrations were quantified spectrophotometrically at 260/280 nm by using NanoDrop spectrophotometer. To verify cDNA production, the RPLP0 gene was utilized.

### Quantitative real-time PCR

Positive results of ELISA underwent verification through PCR analysis. This analysis targeted the Tax and HTLV-1 basic leucine zipper factor (HBZ) viral genes , along with the cellular genes which were CSK, GSK3β, MAP3K14, PLCG1, PTPN6, SLP-76, TAK1 using Gene Runner software and Oligo analyzer, our designed primers for targeted genes were designed. The primer sequences are shown in Table 1. Tax, HBZ and RPLP0 primer sequences were used from our previous research [2]. The RT-qPCR assay was carried out in accordance with the manufacturer's guidelines. The expression of each targeted genes were calculated through dividing relative copy number of the target gene by the relative mRNA copies of RPLP0, yielding normalized expression values for each gene. The comparison of all samples was conducted using RPLP0 expression as the reference gene, with the subsequent calculation of the standard deviation. The amplification process consisted of 42 cycles, involving denaturation at 94 °C for 45 s, primer-specific annealing temperatures outlined in Table 1, and extension for 20 s at 72 °C.

**Table 1:** Designed primers for targeted genes in this study for detection by Real-time qPCR is shown

Gene name	Forward primer	Reverse primer	TM	Amplicon size
CSK	TCGGGTGGAGAAGGGCTACAA	CAGGTGCAGCTCGTGGGTTT	60.6	160
GSK3β	CAAGGACGGCAGCAAGGTGA	TGATGGCGACCAGTTCTCCTGA	60.4	161
MAP3K14	CCAAGACATCCACCGCCAAA	CCCAGACTCCTCCTTGCTCAA	58.8	201
PLCG1	TCTGGCGGAACGGGAAAGT	GCTGGTAGTGCGTGATGAGGT	59.4	126
PTPN6	TCAGTGGGCTGGATGCAGAGA	ACCCTGACGGAGAGCGAGAA	60.6	109
SLP-76	GGGCTGGTCGTCCTTTGAAGA	GGAGGGTGGCGGCTCATAAT	59.7	142
MAP3K7	CGTGGGAGCAGTGTGGAGA	CTGTGGTTGCGGCGATCCTA	59.9	97

### Statistical data analysis

We employed GraphPad Prism and SPSS software for conducting t-tests, Mann–Whitney nonparametric tests, and ANOVA nonparametric tests to assess differences and significance among the studied groups. Plot and graphs were generated for visualizing the results, with significance determined by a P-value less than 0.05. The Real-time PCR output analyzed using the Rotorgen model Q6000. All collected data were organized and included by using the Excel software.

## Results

### Demographic data

20 asymptomatic individuals and 20 healthy subjects were included in this study. The asymptomatic carriers (ACs) group comprised 16 males and 4 females, mirroring the gender distribution in the healthy individuals group. Within the ACs group, the average age for males was 48.45 ± 5.81, ranging from 38 to 58. Conversely, in the healthy group, the average age for females was 48 ± 5.22, with a minimum of 43 and a maximum of 53.

### mRNA expression of ACs and healthy samples

**Fig. 2:**
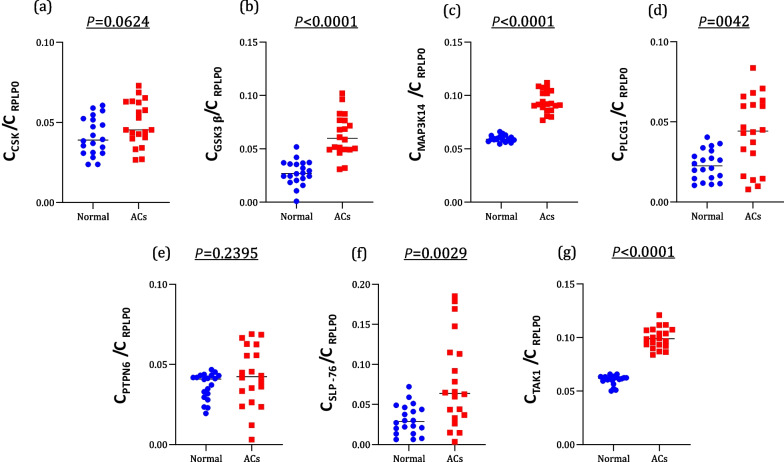
The expression levels of (**a**) CSK, (**b**) GSK3β, (**c**) MAP3K14, (d) PLCG1, (**e**) PTPN6, (**f**) SLP-76, (**g**) TAK1 in ACs patients compared to healthy (normal) group

Primary aim of this study was to investigate the mRNA expression associated with HTLV-1 and its probable relevance in T-cell activation (Fig. 2).

The mean expression of CSK mRNA in patients with ACs was 0.049 ± 0.013 compared to normal group which was 0.041 ± 0.011. While the ACs patients exhibited higher CSK gene expression compared to the normal group, this variance did not demonstrate statistical significance (95% CI, *P* = 0.624) (Fig. 2a).

Regarding GSK3β, the average expression in ACs patients was 0.062 ± 0.019, whereas in the healthy group, it was 0.027 ± 0.011. This difference was statistically significant (95% CI, *P* < 0.0001) (Fig. 2b).

In accordance to MAP3K14, The mean expression in the ACs group was 0.094 ± 0.010, significantly higher than the normal group with a mean of 0.059 ± 0.002 (*P* < 0.0001, CI 95%) (Fig. 2c).

A significant difference was observed in PLCG1 mRNA expression between the ACs group (mean: 0.043 ± 0.022) and the normal group (mean: 0.023 ± 0.009) (*P* = 0.042, CI 95%) (Fig. 2d).

Regarding PTPN6 mRNA expression, the mean in ACs were 0.042 ± 0.018, and in normal subjects, it was 0.036 ± 0.008. However, this difference was not significant (*P* = 0.239, CI 95%) (Fig. 2e).

The ACs patients showed a notably higher average SLP-76 mRNA expression of 0.077 ± 0.056, which was significantly elevated compared to the normal group averaging 0.030 ± 0.018 (*P* = 0.0029, CI 95%). Furthermore, the mean expression of TAK1 mRNA in the ACs group (0.099 ± 0.009) was significantly increased compared to the normal group (0.060 ± 0.004) (*P* < 0.0001, CI 95%) (Fig. 2f and g).

According to our previous research, which was on same samples of this study, the mean expression for Tax was 0.72 ± 0.22 with a maximum of 0.40 and minimum of 0.42 For HBZ, mean expression was 0.65 ± 0.21, with maximum expression of 1.09 and minimum of 0.42 (Fig. 2).

### Investigating connections and trends within measured gene expression

Table 2 shows correlation between studied genes, Tax and HBZ of the HTLV-1. There is significant negative correlation between Tax and GSK3β (*P* < 0.001). This shows that upon upregulation of the Tax, downregulation of the GSK3β can be expected. Also, regarding Table 2, there is negative correlation between MAP3K7 and CSK. Also, positive correlation between MAP3K7 and PTPN6 is seen. While there is some significant correlation between Tax and mentioned genes, there is no significant correlation between HBZ and T-cell activation studied genes.

**Table 2:** Correlation and significance (*P-value*) of targeted genes within the study

Gene	Correlation and *P*-value	CSK	GSK3β	MAP3K14	PLCG1	PTPN6	SLP-76	MAP3K7	Tax	HBZ
CSK	Correlation	1	.254	− .277	− .126	− .156	.215	− .459*	− .254	.OO8
	P-value		.280	.238	.596	.510	.363	**.042**	.280	.975
GSK3β	Correlation		1	− .180	− .122	− .131	− .104	− .368	− .680**	.048
	*P-value*			.446	.609	.582	.663	.110	** < .001**	.840
MAP3K14	Correlation			1	− .134	− .098	− .179	.062	.250	− .146
	*P-value*				.574	.682	.450	.796	.289	.539
PLCG1	Correlation				1	.487*	.344	.263	− .101	.125
	*P-value*					**.029**	.137	.262	.673	.600
PTPN6	Correlation					1	.183	.565**	− .078	.281
	*P-value*						.439	**.009**	.743	.230
SLP-76	Correlation						1	.051	− .138	.257
	*P-value*							.830	.561	.274
MAP3K7	Correlation							1	.089	.343
	*P-value*								.710	.139
Tax	Correlation								1	.083
	*P-value*									.729
HBZ	Correlation									1
	*P-value*									

^*^At the 0.01 level, correlation is significant

^**^At the 0.05 level, correlation is significant

## Discussion

In the context of T-Cell activation and HTLV-1, studies are very limited. Various proteins play a role in inducing T-cell activation, the most important of which are CSK, GSK3β, MAP3K14, PLCG1, PTPN6, SLP-76, TAK1, which were evaluated for mRNA expression in our study. The results of the study indicated the possible induction of increased expression of these genes by HTLV-1. The results showed that virus-infected cells may have a significant increase in the expression of GSK3β, MAP3K14, PLCG1, SLP-76 and TAK1 genes in comparison to healthy population. In contrast, there is no significant changes in PTPN6 and CSK which are negative regulator of T-cell activation in ACs individuals compared to healthy subjects. In general, it seems that activation and proliferation of T lymphocytes is an important part of HTLV-1 pathogenesis.

Modifications were commonly detected in the Tax-interactome and pathways related to T-cell activation. These alterations encompassed NFκB signaling, trafficking, and immunosurveillance pathways [19]. Therefore, irregular activation by Tax within T-cells could potentially hold a significant role in Tax-mediated T-cell activation and transformation [20]. Moreover, some studies indicated that γc-cytokines, specifically IL-2 and IL-15, are generated by CD4 T cells infected with HTLV-1 through transcriptional activation by Tax. Subsequently, these cytokines activate CD8 T cells, leading them to adopt an inflammatory T cell phenotype [21, 22].

Tax, which is one of the most important protein of the HTLV-1 in the context of its pathogenesis, functions as a viral transcription activator protein and plays major role in modulating the expression of cellular genes, particularly in the proliferation of T-cells. This modulation occurs primarily through the activation of the AP-1 and NF-κB. Cells expressing Tax exhibit the ability to circumvent cell-cycle checkpoints, influencing processes related to DNA damage response (DDR) and apoptosis [23]. Consequently, these cells experience genetic and epigenetic alterations, along with modifications in RNA stability [23]. Hence, the activation of signaling pathways by Tax seems to play a role in the initial phases of transformation. Transformed cells subsequently maintain certain constitutively activated pathways crucial for the proliferation and resistance to apoptosis observed in ATL cells [24, 25]. Moreover, there are additional proteins that some studies suggest play roles in T-cell activation. Recent research emphasizes the significance of accessory proteins including p12I, p27I, p13II, and p30II in viral replication and maintaining expression levels in vivo [26]. These proteins not only impact viral infectivity but also enhance T-lymphocyte activation and have the potential to influence gene transcription and mitochondrial function. Specifically, p12I activates NFAT, a crucial T cell transcription factor, while p30II regulates cellular gene expression (26).

C-terminal Src kinase (CSK), a cytoplasmic tyrosine kinase plays a major role in overseeing cellular signal transduction, especially in activation of T-cells. CSK is recognized for its capability to negatively modulate the activity of Src family kinases (SFKs), which contribute to diverse signaling pathways. During T-cell activation, CSK functions as a regulator by phosphorylating a specific tyrosine residue (Tyr-527 in the case of c-Src) within the C-terminal tail of SFKs. This phosphorylation event induces a conformational change that inhibits SFKs' kinase activity, leading to the suppression of their function. [27–31]. An investigation was conducted on C-terminal Src kinase (CSK) within the context of its involvement in T-cell activation and T-cell receptor (TCR) signaling. The research suggests that the presence of the adaptor protein TNF receptor-associated factor 3 (TRAF3) is essential for effective TCR signaling and the normal functioning of T cells. The study, using TRAF3-deficient mouse and human T cells, observed a significant decrease in the activating phosphorylation of the TCR-associated kinase Lck [32]. According to the findings, TRAF3 plays a role in inhibiting Lck inhibitors, such as C-terminal Src kinase (CSK). TRAF3 forms an association with CSK, facilitating its dissociation from the plasma membrane, and it regulates the TCR/CD28-induced localization of another inhibitor, protein tyrosine phosphatase N22 (PTPN22). Loss of TRAF3 leads to elevated levels of CSK and PTPN22 in fractions of T cell membrane. This underscores the significance of TRAF3 in enhancing T cell activation by overseeing the localization and functions of early TCR signaling inhibitors, including CSK [32]. A separate investigation focused on the inhibitory receptor LAIR-1 and its role in suppressing T-cell activation by regulating C-terminal Src kinase (CSK). Contribution of LAIR-1 is to inhibit T-cell receptor signaling by diminishing the phosphorylation of essential components such as LCK, LYN, ZAP-70, and mitogen-activated protein kinases. The intracellular section of LAIR-1, which includes immunoreceptor tyrosine-based inhibition motifs, binds to CSK [33]. This study posits that CSK plays a pivotal role in the LAIR-1-induced inhibition of human TCR signal transmission, suggesting the activation of LAIR-1 as a potential strategy for managing inflammation and addressing autoimmune conditions [33].

Regarding our study, we have seen upregulation of CSK in the ACs compared to healthy individuals, but this upregulation was not significant. Also, negative correlation between Tax and CSK is seen which may explain its non-significant result compared to other T-cell activation associated genes.

GSK3β, or Glycogen Synthase Kinase 3 Beta, is a serine/threonine protein kinase crucial in cellular processes, including T-cell activation. It modulates TCR signaling and, upon engagement, its phosphorylation regulates downstream events. GSK3β 's activity impacts IL-2 production, a key cytokine in T-cell responses. The kinase also influences T-cell differentiation into subsets like Th1 and Th2, affecting the balance of inflammatory responses [34]. Its interaction with beta-catenin in the Wnt pathway further influences T-cell development. Dysregulation of GSK3B is linked to immune disorders. Overall, GSK3β plays a multifaceted role in regulating T-cell functions, ensuring proper immune function and response, and maintaining immune system balance. A study explores the use of GSK3 inhibition in GBM-specific CAR-T cells, demonstrating that pharmacologic inhibition of GSK3 enhances T cell proliferation, reduces exhaustion, and promotes a CAR-T effector memory phenotype. This approach resulted in complete tumor elimination in GBM-bearing animals, suggesting that GSK3 inhibition could be a valuable strategy to improve CAR-T immunotherapy outcomes in solid tumors like GBM [34]. Also, another study discovered that GSK3 beta regulates PDL1 levels suggests a potential impact of inhibiting GSK3 beta on the immune response against tumors [35, 36]. In the context of HTLV-1 infection, A study reported the use of the GSK3β inhibitor, 9-ING-41, in treating ATLL. GSK3β, which implicated in tumorigenesis, was inhibited by 9-ING-41, leading to a durable response in a patient with refractory ATLL. The treatment resulted in a partial response, sustained reduction of ATLL cells, and increased secretion of immune-related molecules by CD8 + T cells [37]. Also, there are various other proteins which have positive effect on T-cell activation. A research investigated the the effect of PLCG1 mutations, particularly S345F, in ATLL. The S345F mutation increases basal PLC activity, contributing to cell proliferation, aggregation, and chemotaxis. Reverting the mutation reduces PLC activity and associated cellular functions in ATL-derived cells [38]. In addition, in accordance to HTLV-1 infection, PLCG1 encodes phospholipase C γ-1 (PLC-γ1), a crucial component in early T-cell receptor (TCR) signaling. Upon TCR activation, PLC-γ1 undergoes tyrosine phosphorylation, initiating the production of two second messengers: inositol 1,4,5-trisphosphate (IP3) and diacylglycerol (DAG). Subsequently, IP3 mobilizes intracellular calcium, crucial for NFAT activation, while DAG stimulates protein kinase C (PKC), facilitating NF-κB signaling [39]. MAP3K14 is involved in the activation of the NF-κB signaling pathway, which plays a role in T-cell activation and immune responses. It participates in the regulation of gene expression and cytokine production. Some studies reported that MAP3K14 is overexpressed in HTLV-1 infected cells and especially in ATLL cells. Overexpression of MAP3K14 leads to T-cell activation [23, 40].

In our study, we have also seen upregulation of GSK3β and PLCG1 which both contribute to T-cell activation and facilitate it. Both upregulations are reported significant despite the negative correlation between Tax and GSK3β. This may show that upregulation of mentioned genes are associated with T-cell activation in ACs compared to healthy individuals.

SH2 domain-containing leukocyte protein of 76 kDa (SLP-76) holds significant importance in T-cell activation. SLP-76 acts as a crucial adaptor protein pivotal in T-cell receptor (TCR) signaling. Its phosphorylation occurs upon TCR engagement, serving as a scaffold for various signaling molecules. This function aids in the activation of downstream signaling pathways which are important for T-cell activation and immune responses. Therefore, SLP-76 plays a facilitative role in T-cell activation, involving ACK1 in its mechanisms [41, 42]. Following our study, we have seen an increased mRNA expression of SLP-76 which may be associated with facilitation in T-cell activation. Virus may use this mechanism for its increased replication and numbers.

PTPN6 is a negative regulator of T-cell receptor signaling. It dephosphorylates and inhibits various signaling molecules, acting as a key player in maintaining the balance of T-cell activation and tolerance. Loss of PTPN6 is showed to be linked with higher NF-KB activation in T-cell lymphoma [43]. Additionally, research has demonstrated that brief STAT3 phosphorylation and activation play a crucial role in immune cell development, including T cells and B cells, as well as functions such as cell growth, proliferation, migration, and death. However, persistent and overactive STAT3 is associated with inflammation, heightened proliferation and survival of cells, increased angiogenesis, metastasis, and disruption of immunity against cancerous cells. PTPN6 acts as a destabilizer of STAT3, resulting in reduced T-cell activation and proliferation [44].

Following our study, there is no significant upregulation of PTPN6 in ACs. This shows that, PTPN6 which acts as T-cell activation suppressor, has no significant changes compared to other genes associated with the activation of this pathway. Also, negative correlation between PTPN6 and Tax is seen.

MAP3K7 (also known as Transforming growth factor-beta-activated kinase 1 or TAK1) is also involved in the activation of T-cells by its contribution in the TGF-β signaling. It can activate downstream kinases, influencing gene expression and cellular responses. An article discussed cellular drug resistance as a major hurdle in cancer chemotherapy, emphasizing the association of chemoresistance with alterations in key pathways such as the MAPK pathway. It indicates TAK1 as a central player, acting as a hub for converging IL1 and TGF-β signaling. TAK1 regulates the phosphorylation and activation transcription factors, mediating inflammatory and pro-survival responses. An article focused on the probable therapeutic effect of targeting TAK1, especially in tumors exhibiting chemoresistance and Epithelial to Mesenchymal Transition (EMT) [45–47]. Efforts to design TAK1 kinase activity inhibitors have been made, showing efficacy in preclinical studies when combined with conventional chemotherapeutic drugs. The article covers TAK1 regulation mechanisms and the development of small molecule inhibitors, emphasizing TAK1 as a potential target for improving tumor responses to anticancer therapies. Furthermore, there are studies indicating that TAK1 augments NF-κB activation induced by TGF-β. This is supported by observations where the knockdown of TAK1 through siRNA resulted in a decrease in TGF-β1-induced phosphorylation of IKK, IκB, and RELA. Additionally, there was a reduction diminished transcription of NF-κB-induced reporter and target genes [45–49]. In the context of our study, an increased expression in TAK1 were seen which may be correlated with T-cell activation following HTLV-1 infection in ACs.

Our study also has some limitations. Upregulation of mentioned mRNAs in T-cell activation needs to be confirmed by western blotting. In addition, this study should be conducted on a higher population scale in order to generalize the results to the entire ACs population. One of the other limitations of this study is the lack of access to patients with HAM/TSP and ATLL to compare whether the expression level in these two groups is as high as ACs or it is much different from this group. However, our study provides valuable insights into mRNA expression level changes within the ACs compared to healthy individuals which results in better insights into HTLV-1 pathogenesis.

## Conclusion

Our study delved into the intricate landscape of T-cell activation in the context of HTLV-1 infection, focusing on mRNA expressions of key regulatory proteins. The investigation revealed significant upregulation of GSK3β, MAP3K14, PLCG1, SLP-76, and TAK1 in ACs compared to healthy subjects, suggesting a probable association between HTLV-1 infection and the activation of these T-cell signaling pathways.

The observed differential mRNA expressions point towards a potential role of HTLV-1 in orchestrating molecular events conducive to T-cell activation. While CSK and PTPN6 showed no significant changes, indicating no forceful suppression, their roles as negative regulators might be overridden by the overall heightened activation observed in ACs. This subtle imbalance in T-cell regulatory mechanisms could contribute to the virus's ability to persist and establish reservoirs within the host. In summary, our investigation contributes to the limited understanding of the multifaceted dynamics between HTLV-1 and the host immune system. The identification of specific mRNA expression patterns associated with T-cell activation in asymptomatic carriers not only enhances our understanding of HTLV-1 pathogenesis but also opens avenues for probable targeted therapeutic interventions. Future studies should explore the functional implications of these mRNA expression changes and their potential as early markers for disease progression in HTLV-1-infected individuals.

## Data Availability

Data will be made available on reasonable request.
